# Severe Myocardial Bridging Without Obstructive Coronary Artery Disease Presenting as Non-ST-Elevation Myocardial Infarction: A Management Challenge in Sinus Bradycardia

**DOI:** 10.7759/cureus.115159

**Published:** 2026-08-25

**Authors:** Branco G Bettinotti, Jose G Gomez, Koushik Sanku, Viky Loescher, Esteban Escolar

**Affiliations:** 1 Department of Internal Medicine, Mount Sinai Medical Center, Miami, USA; 2 Semyon and Janna Friedman Advanced Research Institute, Mount Sinai Medical Center, Miami, USA; 3 Division of Cardiology, Mount Sinai Heart Institute, Mount Sinai Medical Center, Miami, USA; 4 Department of Radiology, Mount Sinai Medical Center, Miami, USA

**Keywords:** adrenergic beta-antagonists, bradycardia, coronary angiography, myocardial bridging, non-st-elevation myocardial infarction

## Abstract

Myocardial bridging (MB) is an anomaly in which a segment of a coronary artery takes an intramyocardial route. It can increase the risk of cardiovascular complications. Beta-blockers (BB) are usually the first line of treatment, and management may be challenging in patients with bradycardia.

A 53-year-old male former smoker with hypertension and dyslipidemia presented to the emergency department complaining of retrosternal chest pain that awakened him from sleep and dyspnea. Upon evaluation, an electrocardiogram (ECG) revealed sinus bradycardia with T-wave inversions in leads II, III, aVF, and V3-V6. High-sensitivity troponin peaked at 335 pg/mL (4-79 pg/mL), and the patient was admitted with a diagnosis of non-ST-elevation myocardial infarction (NSTEMI). He was given 324 mg of aspirin, a heparin drip was started, and pain improved after sublingual nitroglycerin. Transthoracic echocardiogram (TTE) demonstrated a preserved left ventricular function without wall motion abnormalities. Coronary angiography identified a MB in the mid-left anterior descending coronary artery (LAD) without atherosclerotic disease. Coronary CT angiography described a 20 mm long and 7 mm deep MB with 90%-99% dynamic obstruction during end-systole. After a Heart Team meeting, the decision was to attempt medical management with metoprolol as tolerated. The patient remained angina-free and asymptomatic. A stress test prior to discharge was negative for ischemia or recurrent pain. After one-year follow-up, he has remained asymptomatic, with repeated unremarkable stress tests after reduction of BB dose.

Severe MB may be associated with significant cardiovascular complications, including NSTEMI. Treatment poses a challenge in patients with bradycardia. This case illustrates that cautious beta-blocker therapy may be a feasible option in selected patients.

## Introduction

Myocardial bridging (MB) is an anomaly in which a segment of a coronary artery, described as a tunneled artery, takes an intramyocardial route [1,2]. The prevalence of MB is conservatively estimated to be between 21% and 25%, depending on diagnostic modality [2]. Among cases, around 60% occurred in men [2]. Coronary computed tomography angiography (CCTA) is a non-invasive imaging modality that allows for precise anatomical identification of lesions [2]. Although often considered benign, this common variant has been linked to an increased risk of cardiovascular complications, and in a subset of patients presenting with acute coronary syndrome, unstable angina was the most likely presentation [1]. In cases presenting with myocardial infarction (MI) in the absence of coronary obstruction of 50% or more, it is important to consider myocardial infarction without obstructive coronary disease (MINOCA) and investigate for its components [3]. The presence of MB has not been clearly associated with a significantly higher risk of MI [4]. Physiologically, 85% of coronary flow occurs during diastole and 15% during systole. When systolic compression of the MB exceeds 75%, some degree of compression can extend to diastole, compromising maximal coronary flow and delaying myocardial relaxation, contributing to myocardial ischemia and manifesting as angina [1,2]. Management with beta-blockers (BB) or non-dihydropyridine calcium channel blockers (CCB) is typically the initial approach to treatment in symptomatic patients due to the benefit from negative chronotropic effect optimizing diastolic coronary filling, with more invasive procedures such as myotomy and bypass surgery reserved for refractory cases [5,6]. Management of patients with underlying bradycardia can pose a clinical challenge. We present a case of non-ST-elevation myocardial infarction (NSTEMI) occurring in the setting of severe myocardial bridging without obstructive coronary disease, cautiously managed with BB despite baseline sinus bradycardia.

This article was previously presented as a poster at the 2026 ACP Florida Chapter Annual Residents and Medical Students Meeting in March 2026.

## Case presentation

**Figure 1:**
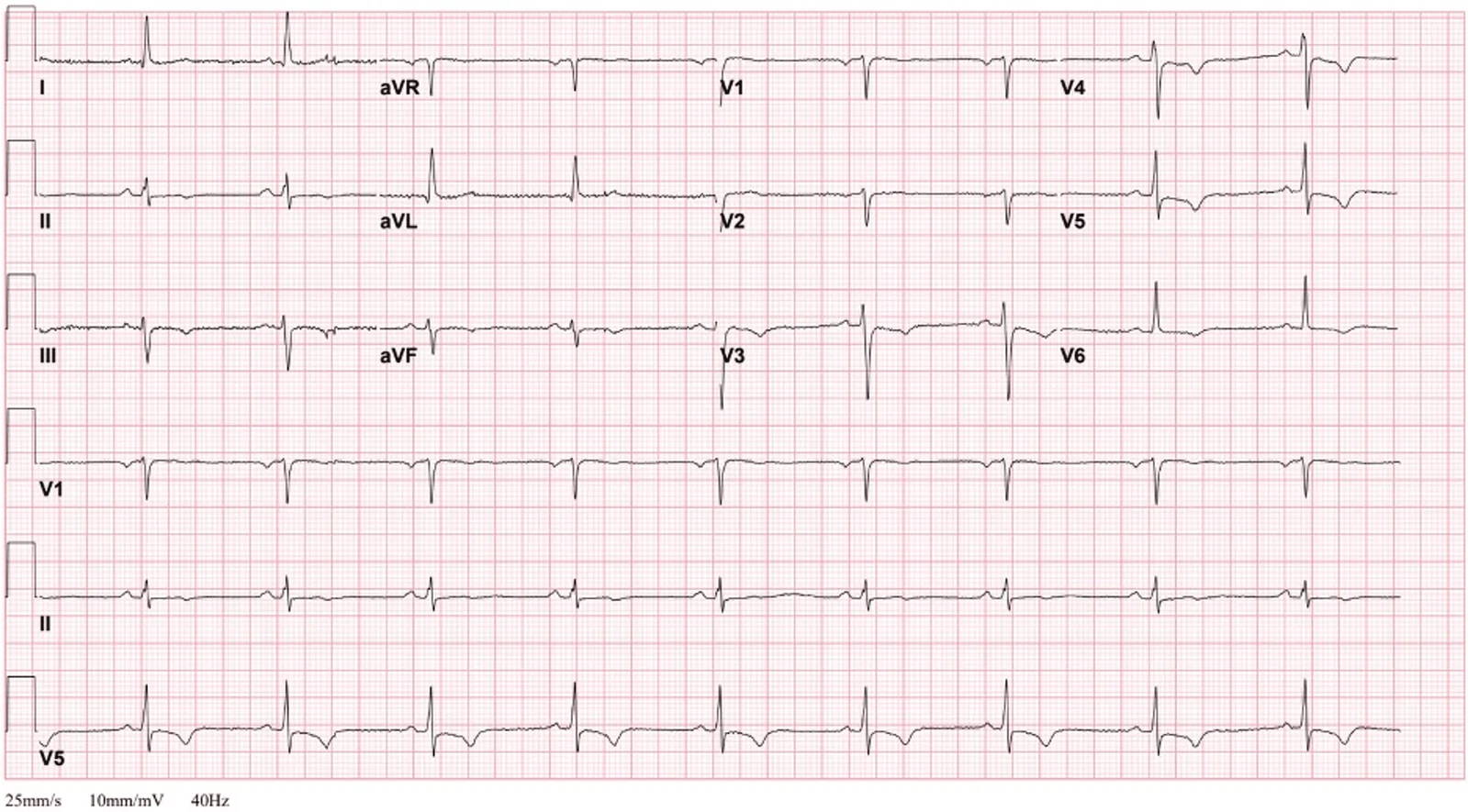
ECG on admission Sinus bradycardia (heart rate: 56 bpm) and T-wave inversions in V3-V6, II, III, and aVF. ECG: electrocardiogram

A 53-year-old male former smoker with hypertension and dyslipidemia presented with sudden-onset, oppressive, non-radiating retrosternal chest pain that awakened him from sleep and was associated with shortness of breath. He denied diaphoresis, palpitations, and illicit substance use. Aside from bradycardia at 55 bpm, vital signs were unremarkable on admission. An electrocardiogram (ECG) revealed sinus bradycardia and new T-wave inversions on leads II, III, aVF, and V3-V6 (Figure 1). Troponins were elevated and peaked at 334.9 pg/mL (4-79 pg/mL), identifying an NSTEMI. Pain improved significantly with sublingual nitroglycerin. He was loaded with 325 mg of oral aspirin and started on a heparin drip.

**Figure 2:**
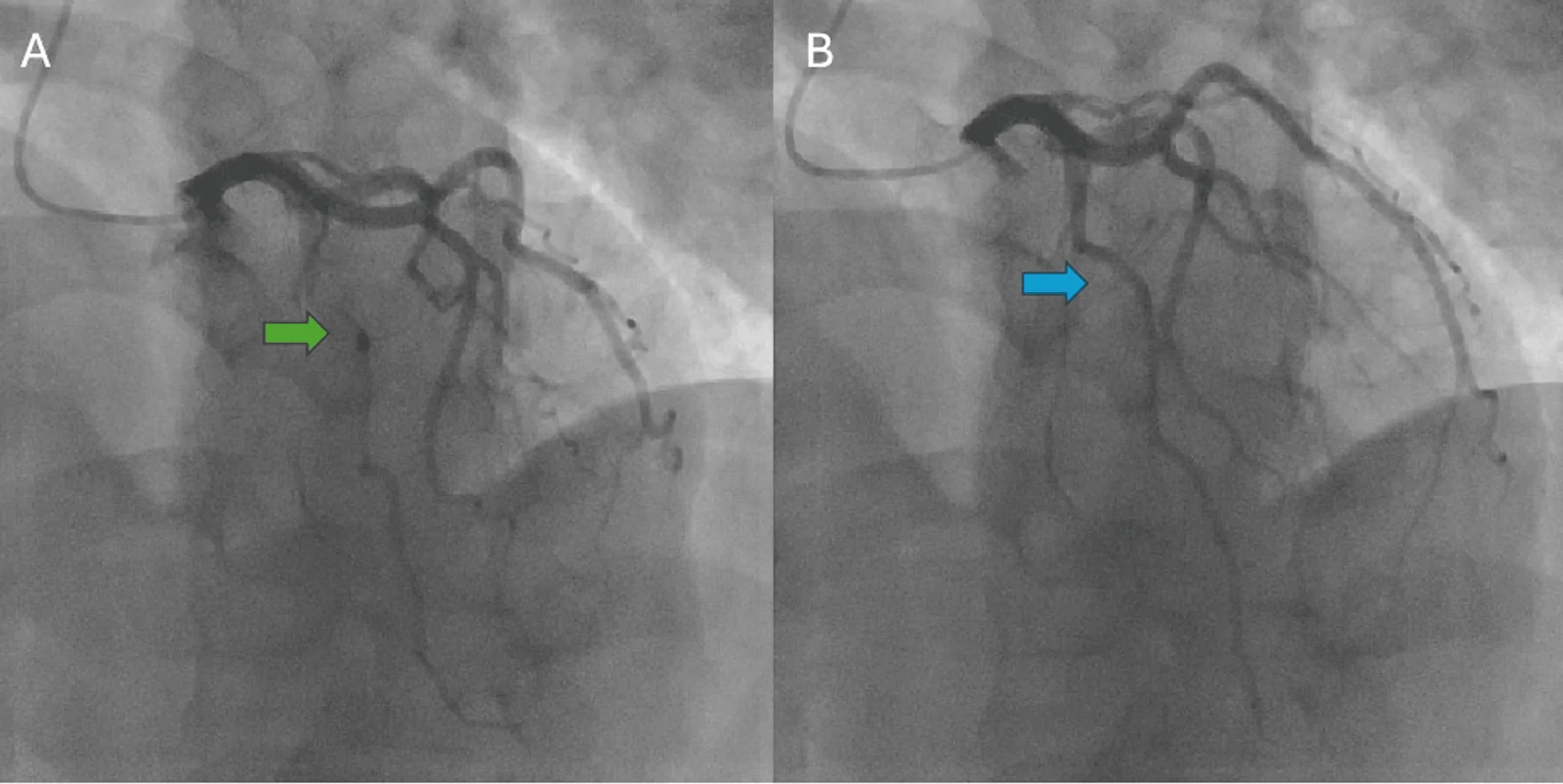
Conventional coronary angiography Conventional coronary angiography revealing a myocardial bridge of the LAD. Significant obstruction (>75%) of the mid-LAD is depicted only during systole (A, green arrow) and adequate filling during diastole (B, light blue arrow). LAD: left anterior descending coronary artery

**Figure 3:**
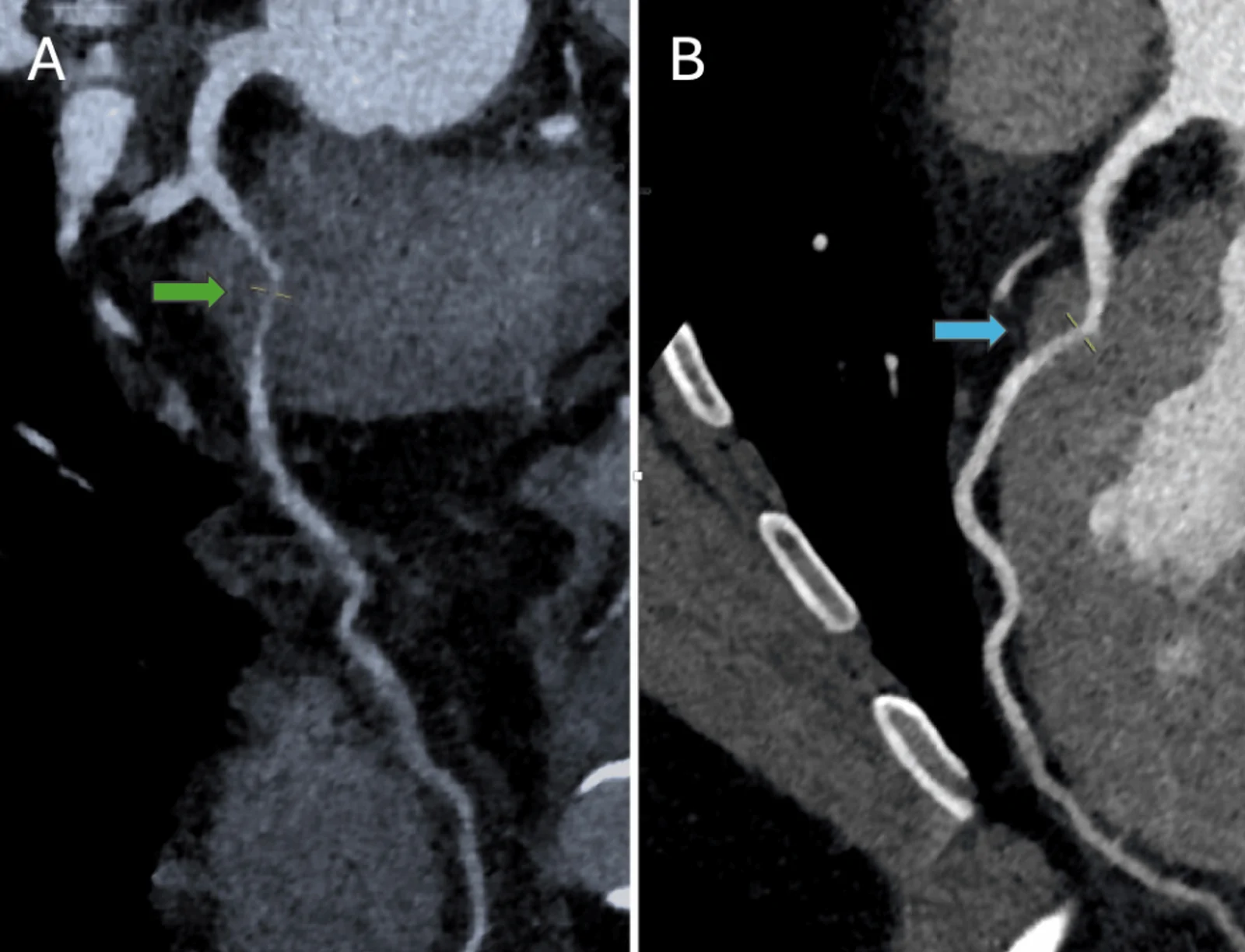
Coronary computed tomography angiography images Curved planar reformation technique revealing a 20 mm long and 7 mm deep myocardial bridge of the mid-LAD, with luminal obstruction of at least 75% during systole (A, green arrow) and adequate filling during diastole (B, light blue arrow). LAD: left anterior descending coronary artery

A transthoracic echocardiogram (TTE) demonstrated an ejection fraction (EF) of 55%-60% with normal diastolic function and no wall motion abnormalities. Conventional coronary angiography (CCA) was performed within 24 hours of admission, which identified MB of the mid-left anterior descending coronary artery (LAD) and some irregularities in the non-MB portion of the LAD without significant obstructive disease (Figure 2). Intra-arterial nitroglycerin was given, without change in the acquired coronary images (Video 1 and Video 2). No intracoronary imaging or spasm provocation testing (SPT) were performed. Coronary computed tomography angiography (CCTA) was performed to identify the tunneled artery further, revealing a 20 mm long and 7 mm deep MB, with 90%-99% obstruction only during end-systole. No proximal atherosclerotic plaque was identified by CCA or CCTA (Figure 3). Cardiac magnetic resonance (CMR) was not performed due to the patient's preference.

**Video 1:** Conventional coronary angiography Right anterior oblique view revealing left anterior descending myocardial bridge.

**Video 2:** Conventional coronary angiography Right anterior oblique view with myocardial bridge on the left anterior descending artery.

**Figure 4:**
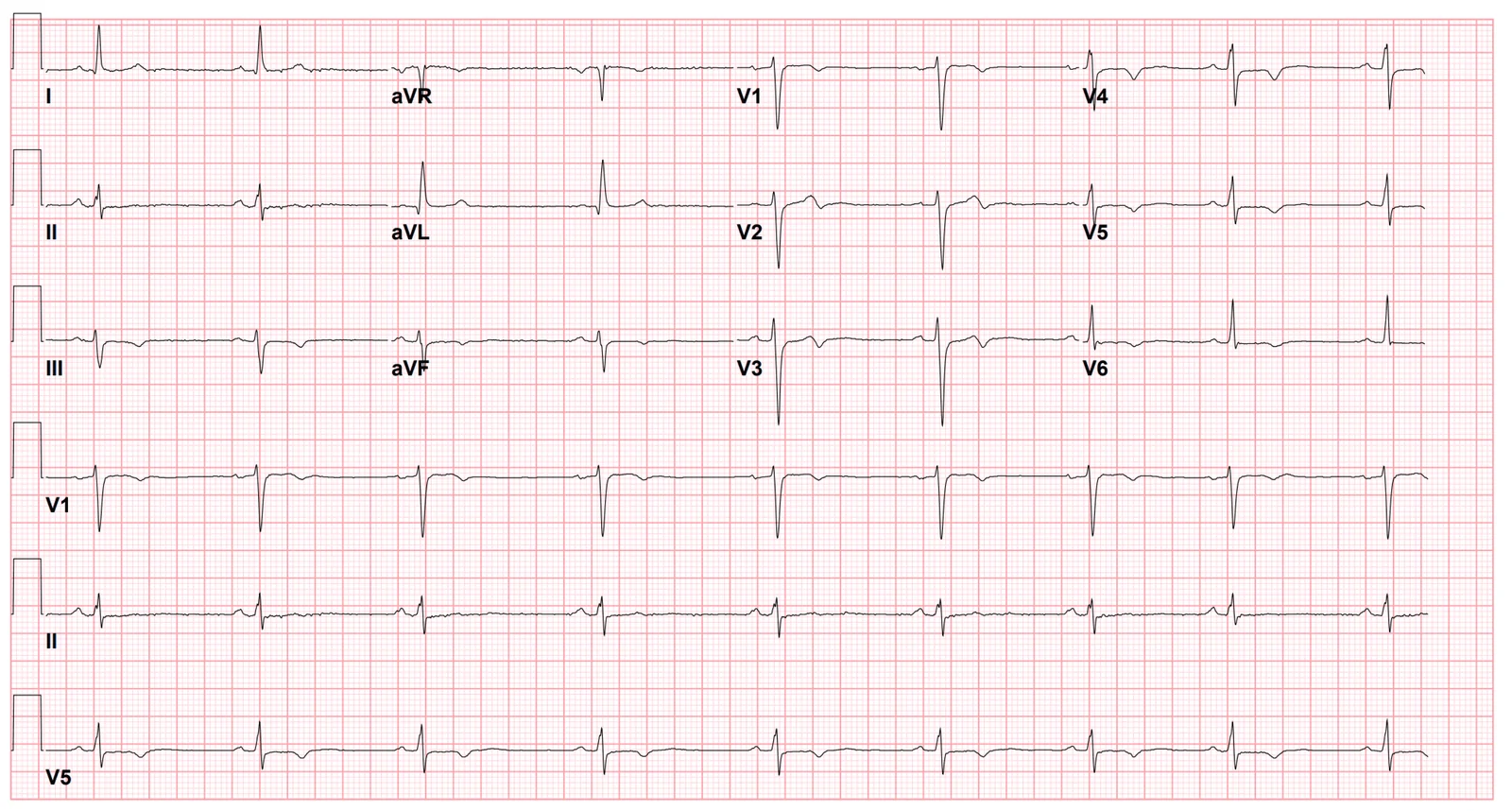
Repeat ECG after beta-blocker therapy Sinus bradycardia (heart rate: 52 bpm) and unchanged T-wave inversions in V3-V6, II, III, and aVF. ECG: electrocardiogram

After a Heart Team meeting between clinical and interventional cardiology, and cardiothoracic surgery, the decision was to attempt medical management with metoprolol succinate 50 mg daily, which he tolerated well throughout admission, with the lowest heart rate (HR) being 50 bpm. Multiple ECGs were repeated during observation, without significant changes (Figure 4). A treadmill exercise stress test was performed on day 6, achieving 13.4 METS, Duke treadmill score of 12, explained by 12 minutes of exercise, without chest pain, dyspnea, or ischemic ECG changes. The patient was safely discharged with a plan to continue medical management and close follow-up. At one-year follow-up, he remained pain-free, repeat stress test noted excellent exercise capacity at 13.4 METS, Duke treadmill score of 6.4, explained by 11.4 minutes of exercise, a less than 1 mm ST segment deviation (interpreted as normal ECG response), and lack of chest pain; despite numeric variation between both Duke scores, both of them lay within the low post-test probability for significant obstructive coronary artery disease.

## Discussion

Although myocardial bridging is frequently considered a benign anatomical variant with a favorable prognosis, severe forms may produce clinically significant myocardial ischemia. In a meta-analysis of 21 studies comparing patients with and without MB, MB was associated with higher odds of major adverse cardiac events (OR = 1.52), myocardial ischemia (OR = 3.00), and endothelial dysfunction. No significant difference in cardiovascular death was identified [4]. Prognosis may vary according to clinical presentation, ischemic burden, and anatomical severity.

This case illustrates a clinically relevant dilemma: BB remain commonly used for the management of symptomatic MB, and baseline sinus bradycardia may limit their use, requiring individualized assessment. In this patient, cautious BB therapy was tolerated under close monitoring and was associated with sustained symptom resolution at one year.

Multiple classifications for MB have been proposed. By depth, lesions can be classified as superficial when they are 1-2 mm and deep if they are over 2 mm deep in the myocardium [1]. MB is defined as long when the bridged segment is 25 mm or longer [1]. Schwarz et al. described three groups in symptomatic patients: type A without objective signs of ischemia, type B with objective signs of ischemia on non-invasive stress testing, and type C for cases with altered intracoronary hemodynamics on quantitative coronary angiography, coronary flow reserve, or intracoronary Doppler [5]. By degree of systolic narrowing, lesions are classified as class I (<50%), class II (50%-75%), or class III (>75%) [2].

Myocardial ischemia in MB can be attributed to coronary narrowing during systole. Deeper and longer lesions have more severe obstruction, and in class III lesions, some degree of obstruction may persist during early and mid-diastole. Hypertension, tachycardia, and coronary vasospasm reduce coronary flow, exacerbating symptoms [7]. Nitrates can exacerbate symptoms by causing coronary steal and tachycardia, and are not recommended as a treatment option; however, intra-arterial nitroglycerin did not alter the angiographic findings in our patient [1].

The major limitations of our case are the absence of SPT, intravascular imaging, and CMR. Therefore, vasospasm associated with the bridged segment cannot be excluded as a contributor to his presentation. Although coronary dissection was not identified in the CCTA, lack of intravascular imaging limits its definitive exclusion. Similarly, myocarditis cannot be completely ruled out without a CMR [3]. In the absence of obstructive coronary disease, severe MB was considered the most plausible mechanism of NSTEMI in this patient, supported by CCA and CCTA findings demonstrating a deep, class III lesion in the setting of ischemic symptoms and troponin elevation.

No current guidelines exist for the management of MB. However, medical management with BB or CCB remains the initial recommended approach because these agents lower heart rate, prolong diastole, and thereby improve diastolic flow [1,2]. Surgical approaches such as unroofing may be considered for young patients with superficial bridges that are refractory to medical management. Coronary artery bypass grafting is generally reserved as a last option for deep or long lesions with severe obstruction because of the high risk of competitive flow and graft failure [1,8,9]. It is worth noting that coronary stenting remains controversial due to up to 36% rates of in-stent restenosis seven weeks post-intervention, risk of perforation, and less improvement in symptoms than the other approaches [1,2,10].

## Conclusions

Severe myocardial bridging could present as NSTEMI and should be considered as a potential diagnosis when obstructive coronary lesions are not identified. Multimodality evaluation, including CCTA, can help characterize lesion anatomy and severity to individualize management.

Despite underlying bradycardia, beta-blocker therapy may be feasible in selected patients under appropriate monitoring and close follow-up. This cannot be generalized, and further studies are needed to evaluate further treatment options.
